# Exploring the fibrinolytic potential of marine *Actinoalloteichus caeruleus* isolated from Bay of Bengal coast

**DOI:** 10.1186/s12866-025-03815-w

**Published:** 2025-03-03

**Authors:** Kothari Neeti Suresh, Subathra Devi. C

**Affiliations:** School of Bio Sciences and Technology, Vellore Institute of Technology, Vellore, Tamil Nadu India

**Keywords:** *Actinoalloteichus caeruleus*, Thrombolytic, Fibrinolytic, Marine actinomycetota, Secondary metabolites, Thrombosis

## Abstract

**Background:**

One of the main causes of several cardiovascular diseases that have an elevated mortality rate globally is intravascular thrombosis. The current fibrinolytic enzymes, are quite expensive and have a lot of side effects, thus it is necessary to develop alternate, economical techniques for the low-cost manufacture of these vital enzymes. Microbial fibrinolytic enzymes have the capacity to break up these clots and are relatively cheaper with minimal side effects and quick growth rate. Marine actinomycetota are the most prolific prokaryotes, which are capable of synthesizing novel secondary metabolites and are of industrial importance in pharmaceutical and various other industries. Thus, the objective of the research is to isolate, screen and characterize fibrinolytic protease producing actinomycetota from marine samples.

**Results:**

In this study, 35 actinomycetota have been successfully isolated from marine water and sediment samples. Among them, 12 isolates were protease positive and on secondary screening 5 isolates showed fibrinolytic activity. Out of the 5 isolates, one potent isolate’s clot lysis activity was found out to be 93.12 ± 0.18% and its fibrinolytic potential was determined on fibrin agar plates. Based on the morphological, physiological, biochemical, and molecular analysis, the potent strain (NK60) was identified as *Actinoalloteichus caeruleus*.

**Conclusions:**

In this present study, a rare actinomycetota has been isolated from the Bay of Bengal coast. This is the first study reporting the potent fibrinolytic activity of *A. caeruleus*, isolated from marine water. This clot-busting enzyme has significant pharmacological value in the management of coronary artery diseases. In the near future, *A. caeruleus* can serve as an explicit source for commercial production of fibrinolytic enzymes.

## Background

One of the most prevalent illnesses in modern times is thrombosis. Thrombosis is caused by the buildup of fibrin, when tissue is damaged. Usually, the fibrin formed at the site of injury is broken down by plasmin, however when an embolus is formed within a blood vessel, it blocks the circulation of blood, resulting in the formation of thrombus, causing arterial or venous thrombosis [1]. Fibrinolytic protease are hydrolase type of protein that play a vital role in thrombotic illness by breaking down the fibrin, through activated thrombin resulting in the dissolution of the blood clot [2, 3]. Cardiovascular conditions like coronary thrombosis, veinous thrombosis, pulmonary thrombo-embolism, stroke and arrhythmias are closely linked to thrombosis [4]. Heart related conditions, occupy the first position for highest number of death and the most common causes of death, according to the WHO survey, are heart attacks and strokes. According to the 2019 statistics of WHO, fatalities pertaining to cardiovascular conditions have reached 32% and would result in about 17.9 million dissolution [5, 6]. In the olden days embolism was cured by the use of anti-coagulant like warfarin, heparin but as the understanding of the molecular mechanism underlying the disease became clearer with the advent of knowledge, roles of enzymes in the body also grew clearer. Thus, enzymes hold industrial importance and are a valuable tool [7, 8]. The clinically approved thrombolytic enzymes employed for treating cardiovascular disease are tissue plasminogen activator, streptokinase, urokinase, which will breakdown fibrin by converting plasminogen to plasmin, but all of these enzymes have some drawbacks like high price, shorter half-life, bleeding in the GI tract, allergic reaction, weak binding specificity and re-occlusion [9, 10]. Thus, there is a need to look into novel and reliable thrombolytic medicines as synthetic therapies have detrimental effects.

A substantial amount of interest in medicine has been sparked by microbial fibrinolytic enzymes because of their large diversity, quick growth rate, genetic manipulation susceptibility, ease of production and low cost. The vast diversity of microorganisms raises the possibility of acquiring enzyme of medicinal importance, with varied characteristics [11]. Fibrinolytic enzymes producing microbial strains have been obtained from three distinct groups i.e. bacteria, fungi and actinomycetota and the genus *Bacillus* sp. [12]*, Staphylococcus* sp. [13]*, Pseudomonas* sp.*, Fusarium* sp. [8] and *Streptomyces* sp. [14] are the major producers of fibrinolytic proteases.

The most prolific type of system in terms of both ecology and economy are marine environment, which are abundant in biological diversity. Because of their high nutrient content, they provide a favourable niche for a wide variety of microorganisms. Marine organisms living in such harsh environments, have greatly enhanced their physiological and metabolic capacities and are regarded as reservoirs for a variety of ecologically valuable microbial populations that are abundant in enzymes and bioactive compounds, however are largely underexplored [15].

Fibrinolytic enzymes producing actinomycetota have been isolated from diverse marine environments. From the Bay of Bengal coast, *Streptomyces lusitanus* was isolated which exhibited good fibrinolytic activity [16]. Similarly, from marine soil, a *Streptomyces* sp. was obtained, which showed excellent fibrinolysis [17]. Likewise, another *Streptomyces* sp. was isolated from hot springs, which possessed the ability to breakdown fibrin efficiently and in a shorter time-span [18]. From a brown marine sponge *Agelas conifera*, a fibrinolytic enzyme was procured from *Streptomyces radiopugnans* [19]. *Streptomyces rubiginosus* isolated from the soil samples of South East coast of India, exhibited good fibrinolytic activity [14]. From *Streptomyces venezuelae,* a fibrinolytic enzyme thrombinase was extracted from marine sample [20]. About 114 marine actinomycetota was isolated from South-east coast, out of which 12 strains showed profound thrombolytic activity [21]. From the Amazonian lichens, a *Streptomyces* sp. was isolated which exhibited excellent thrombolytic activity [1]. *Streptomyces* *omiyaensis*, a fibrinolytic serine protease from the trypsin family showed higher fibrinolytic activity than urokinase and nattokinase [22]. From *Streptomyces* sp. XZNUM 00004, a fibrinolytic enzyme SFE1 was obtained, which showed promising thrombolytic activity [23]. Therefore, there is no question that marine environment are home to potent and unique microbes, that will open up the way to future research.

Marine actinomycetota that produce fibrinolytic enzymes have not been thoroughly studied and have only been isolated from a few number of marine sources so far, thus in the current investigation an attempt is made to isolate unique actinomycetota from diverse marine sources, which are capable of producing fibrinolytic enzymes. Therefore, the main objective of this study is to isolate, screen and characterize actinomycetota with fibrinolytic activity.

## Methods

### Sample collection

Fifteen Marine sediments and 15 water samples were collected in 2023 from Bay of Bengal coast (Chennai, Pondicherry, Pichavaram, Rameshwaram and Mandapam). These samples were collected in sterile bags, transported to lab immediately and further stored at 4℃ [24].

### Isolation of fibrinolytic protease producing actinomycetota

The collected sediment samples were allowed to air dry at room temperature, for about 2–3 days, followed by drying in hot air oven at 70 °C to reduce the bacterial load of gram-negative bacteria [25]. After the pre-treatment, one gram of sediment sample was mixed thoroughly with 9 ml of filtered, sterilized seawater. An aliquot of 1 ml was taken, serially diluted to 10^–5^ dilutions and spread plated on Actinomycetes Isolation agar and Starch Casein agar plates. No additional salt was added to the medium, as the media preparation was done in 50% seawater and 50% distilled water to favor the growth of marine actinomycetota. Water samples were also processed similarly [26]. To the medium, 50 µg/mL of Nalidixic acid and Cycloheximide were incorporated to inhibit growth of bacteria and fungi respectively. The plates were incubated for 20 to 40 days at room temperature, until actinomycetota colonies were clearly visible [27–29]. On subculturing, the growth was observed on media plates after 5–6 days.

### Screening for fibrinolytic protease producing actinomycetota

#### Primary screening

Primary screening was carried out to identify protease producing actinomycetota. In this skim milk agar plates were made containing 0.5% Peptone, 0.5% Sodium chloride, 10% Skimmed milk powder and 1.5% agar. The 35 actinomycetota isolates were streaked on skim milk agar plates followed by incubation at room temperature for observing zone of hydrolysis [21].

#### Secondary screening

The protease positive strains from primary screening were further evaluated for fibrinolytic activity. For secondary screening, casein plasminogen well diffusion method was performed and plates were incubated at room temperature to observe the fibrinolytic potential of the isolates [19].

#### Clot Lysis activity

Modified Holmstrom method was used to determine the potent isolates clot-lysis activity [19, 30].

#### Fibrin plate assay

Using the fibrin plate method, the fibrinolytic potential of the crude isolate NK60 was assessed [15].

### Characterization of the isolate producing fibrinolytic protease

Molecular characterization of actinomycetota was carried out using 16S rDNA sequencing. On the basis of morphological and biochemical characterization the potent strain was characterized.

### Morphological characterization

#### Gram staining

Gram staining was performed for the fibrinolytic strain to identify the isolate's gram nature. The organism was observed under 10X, 40X and 100X oil immersion [28].

#### Scanning electron microscopy

The morphology of the spore was observed by examining the isolate using scanning electron microscopy. A 1 cm × 1 cm clean slide was taken and a smear was prepared from one month old plate, air dried, followed by keeping it overnight at 40℃ in the oven [28].

#### Biochemical characterization

Biochemical test like starch, urea, casein, gelatin and tween 20 hydrolysis were carried out [31]. Other biochemical test like citrate utilization test, H_2_S production, catalase, oxidase and nitrate reduction test were also performed [32]. Sodium chloride tolerance test was carried out for the potent isolate at various concentration ranging from 0–15% in Starch casein broth and incubated at 30℃ for a period of about 10 days [33].

#### Molecular characterization

The potent isolate was subjected to genomic DNA isolation using the QIAGEN DNeasy UltraClean Microbial Kit and the fragments of 16S rRNA gene were amplified by the primers 16SrRNA-F-27F (AGAGTTTGATCCTGGCTCAG) and 16SrRNA-R-1492R (CGGTTACCTTGTTACGACTT). The single discrete PCR amplicon band was purified using QIAGEN QIAquick PCR purification kit. PCR was performed under the following conditions: initial denaturation for 3 min at 95℃, followed by 30 s denaturation at 95℃, annealing at 50℃ for 30 s, extension for 72℃ for 45 s and final extension for 3 min at 72℃, with 35 cycles. The sequencing reaction was carried out using an ABI 3730xl Genetic Analyzer. Big Dye ™ Terminator V3.1 kit was used for sequencing. The NCBI's BLAST program was then used to perform a similarity search on the acquired sequence. Using Mega 11 software's neighbour joining approach, a phylogenetic tree was built. Bootstrap analysis was carried out to determine the branching pattern’s reproducibility and the number of bootstrap replications were 1000 [34]. The 16S rDNA sequence has been submitted to GenBank, with the accession ID PQ044827.

## Results

### Isolation of fibrinolytic protease producing actinomycetota

In the present study, a total of fifteen marine water and fifteen sediment samples were collected from several locations of Bay of Bengal coast such as Chennai, Pondicherry, Rameshwaram, Pichavaram and Mandapam.

Actinomycetes isolation agar and starch casein agar were used for the isolation process. A total of 35 actinomycetota strains were obtained from 30 different samples. From Mandapam coastal region, 7 actinomycetota strains were isolated from sediment samples, whereas from Pichavaram 2 actinomycetota strains from water sample. From Pondicherry 9 strains from sediment sample and 1 from water sample, whereas from Chennai 8 strains were isolated from sediment sample and from Rameshwaram 7 strains from sediment sample and 1 from water sample were isolated. Starch casein agar facilitated better growth of actinomycetota as higher number of actinomycetota colonies were observed as compared to actinomycetes isolation agar. When compared to marine water, actinomycetota were more prevalent in marine sediments.

### Screening for fibrinolytic protease producing actinomycetota

**Fig.1:**
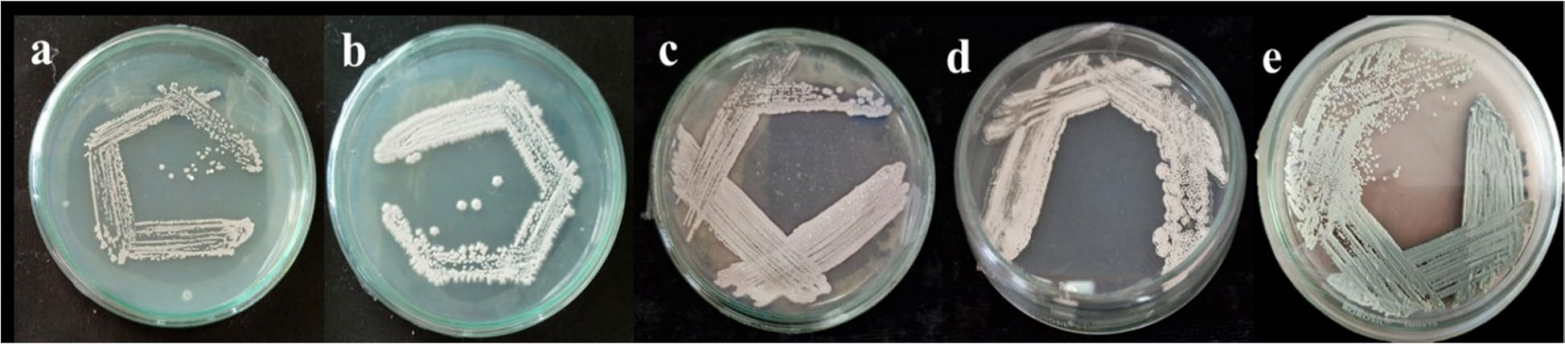
Pure culture of actinomycetota isolated from marine sediments (**a**) NK22 (**b**) NK11 (**c**) NK04 (**d**) NK12 (**e**) marine water *A. caeruleus* NK60

**Fig. 2:**
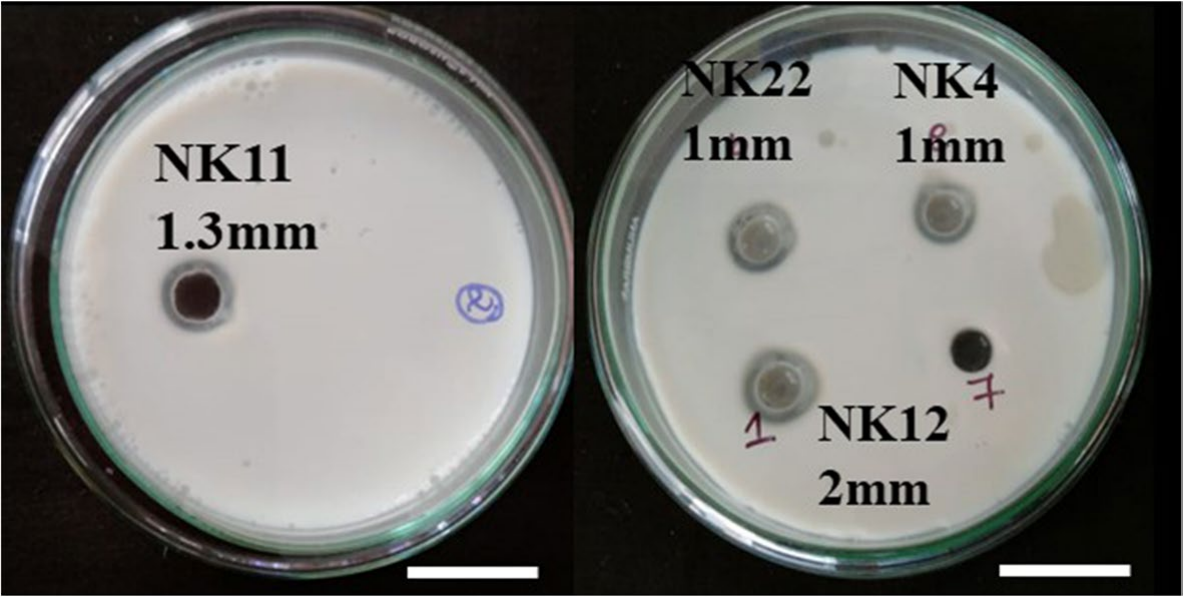
Secondary screening of NK11, NK22, NK04, NK12 (zone of hydrolysis)

**Fig. 3:**
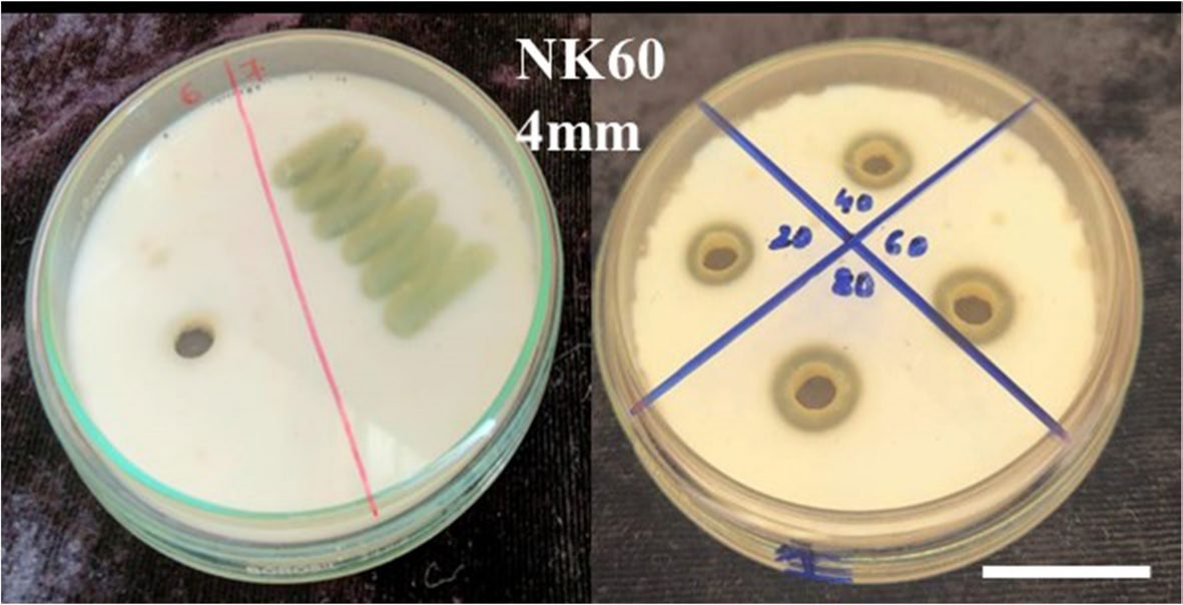
Primary screening and secondary screening for strain NK60 (zone of hydrolysis)

Primary screening was carried out to identify the protease activity of the 35 actinomycetota isolates. Out of 35 isolates, 12 isolates were chosen for secondary screening in accordance with the size of the zone shown on the skimmed milk agar plates. Five of the twelve isolates that tested positive for protease showed zone of hydrolysis when tested further for fibrinolytic activity on casein plasminogen plates (Figs. 1, 2 and 3). Among the five isolates, NK60 exhibited promising results representing 4 ± 0.176 mm zone of hydrolysis and was chosen as the potent isolate. This potent strain of the actinomycetota was capable of efficiently hydrolysing the casein plasminogen in the medium, confirming that the isolate is exhibiting proteolytic activity (Table 1).

**Table 1:** Secondary screening for fibrinolytic protease positive isolates

Actinomycetota Isolates	Zone of hydrolysis (in mm)
NK4	1 ± 0.145
NK11	1.3 ± 0.088
NK12	2 ± 0.033
NK22	1 ± 0.115
NK60	4 ± 0.176

**Fig. 4:**
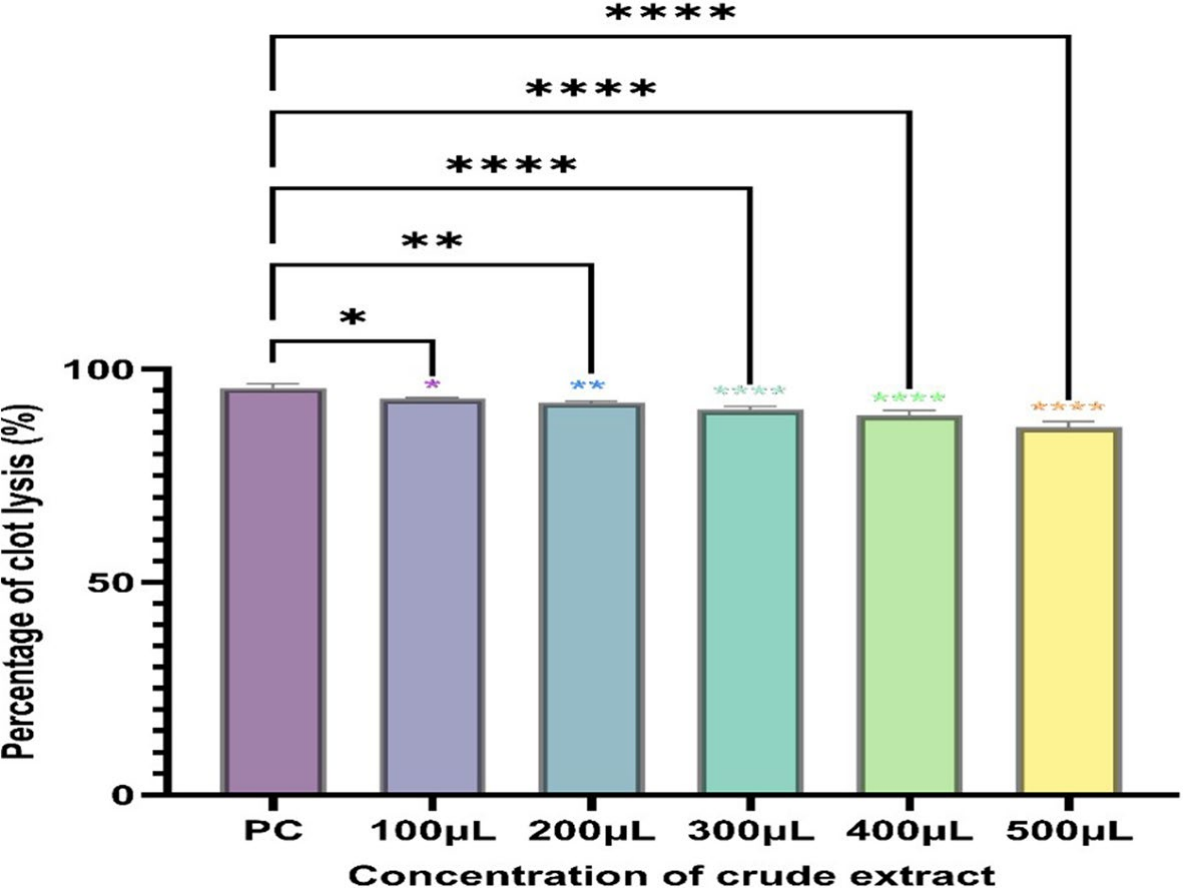
Clot lysis activity of *Actinoalloteichus caeruleus* NK60

**Fig. 5:**
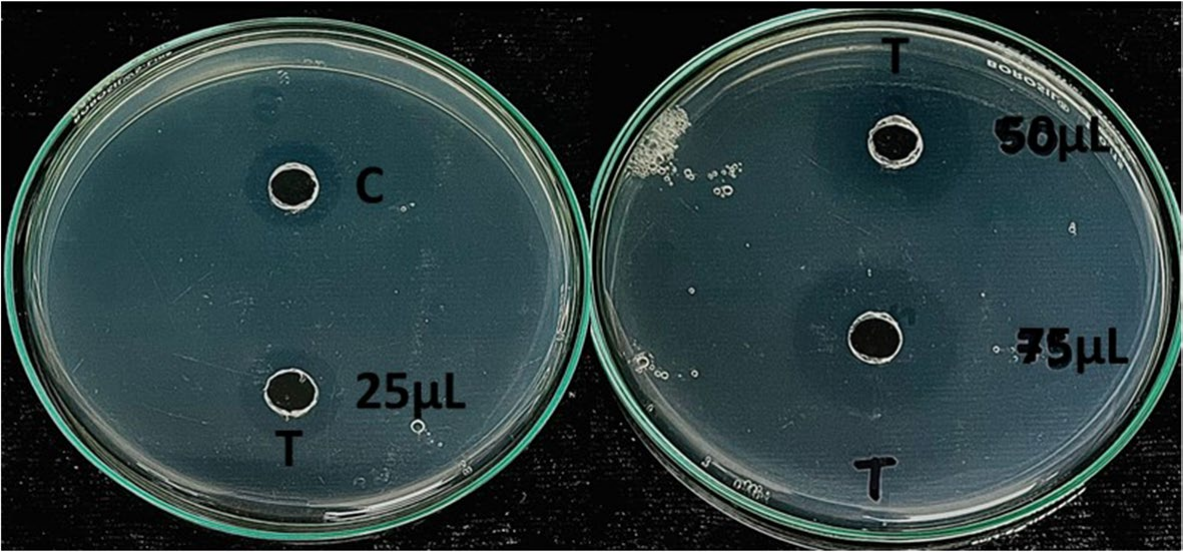
Fibrin plate assay (T- Test sample: crude enzyme from *Actinoalloteichus caeruleus* NK60, C- Control: Standard Streptokinase)

The maximum clot lysis activity of the fibrinolytic protease extracted from the potent isolate NK60 was found to be 93.12 ± 0.18% in 2 h, followed by 91.30 ± 0.52% in 4 h and 88.79% ± 0.76 in 6 h (Fig. 4). On performing one way ANOVA, significant differences were observed between the standard streptokinase and test samples, indicating statistical differences. To determine the fibrinolytic activity of the crude enzyme extract NK60, fibrin plate assay was performed (Fig. 5). The in vitro synthesis of fibrin structure by fibrinogen and thrombin serves as the foundation for the fibrin plate method, resulting in a zone of fibrinolysis around the well. The crude isolate NK60 exhibited promising results representing a 10.6 ± 0.66 mm zone of clearance whereas, the standard Streptokinase showed a zone of 6 mm (Table 2). When compared to standard streptokinase, crude enzyme extract showed a significant fibrinolytic activity.

**Table 2:** Fibrinolytic activity of enzyme extracted from marine *Actinoalloteichus caeruleus* NK60

Samples	Zone of hydrolysis (in mm)
NK60	10.6 ± 0.66
Streptokinase	6 ± 0.57

### Characterization of the isolate producing fibrinolytic protease

**Fig. 6:**
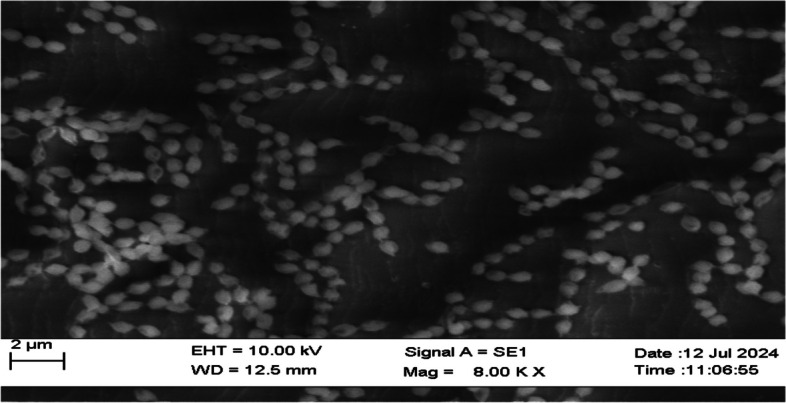
Scanning electron microscope image showing the morphology of *A. caeruleus* NK60 spores

Morphological characterization: Gram staining was used to characterize the morphology of the isolate NK60, revealing that it was a Gram-positive, filamentous, non-motile, spore-forming bacteria. The shape and arrangement of the spores was identified using scanning electron microscopy at various magnifications, displaying a spherical morphology of spore in chains, with warty surface (Fig. 6). On the basis of cultural morphology, five actinomycetota strains were characterized (Table 3).

**Table 3:** Morphological characterization of fibrinolytic protease positive isolates

Isolate number	Morphological characterisation	Pigmented	Gram staining
NK4	Brown powder	Non pigmented	+
NK11	White powdery	Non pigmented	+
NK12	White chalky	Non pigmented	+
NK22	Grey chalky	Non pigmented	+
NK60	White powdery	Blue pigmentation	+

Biochemical characterization: Biochemical characterization was performed for the isolate NK60. The studies depict that the isolate can hydrolyze casein, gelatin, starch and urea. The isolate NK60 showed resistance at 12% w/v NaCl concentration and was able to grow in concentrations of 1% to 11% w/v (Table 4).

**Table 4:** Biochemical Characterization of *A. caeruleus* NK60

Biochemical test	Results
Starch hydrolysis	+
Casein hydrolysis	+
Gelatin hydrolysis	+
Urea hydrolysis	+
Tween 20 hydrolysis	-
Nitrate reduction test	-
H_2_S production test	+
Citrate utilization test	_
Catalase test	_
Oxidase test	_
Salt tolerance test	11% w/v

Positive = ‘ + ’, Negative = ‘-’

**Fig. 7:**
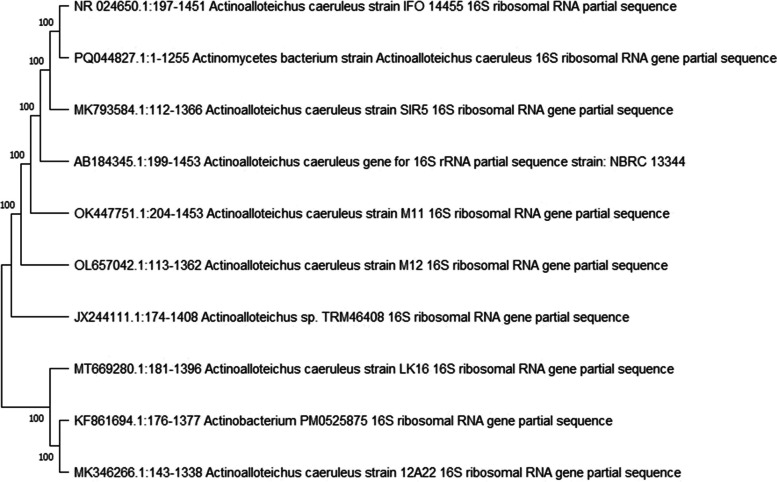
Phylogenetic tree of *A. caeruleus* NK60

Molecular characterization: The 16S rDNA sequence of the strain NK60 showed 100% similarity with *A. caeruleus*. Mega 11 software was used to create the phylogenetic tree. The evolutionary history has been determined using the Neighbour joining method. The sum of branch length of the phylogenetic tree is 31.11203567. Next to the branches is the value of bootstrap test percentage of replicate trees. The expected value was found out to be 0.0 and the query coverage was between 95 to 100%. Maximum composite likelihood method was used for computing the evolutionary distance. Upon analysing the phylogenetic tree, the out-group organism can be identified as KF861694.1 *Actinobacterium* PM0525875 as it belongs to a different genus *Actinobacterium* and other sequences belong to the genus *Actinoalloteichus*. In the final dataset, there was a total of 1027 positions. The 16S rDNA sequence has been deposited to GenBank, with the accession ID PQ044827. (Fig. 7).

## Discussion

Coronary infarction has been the most frequent reason for death and with the adverse side-effects of the current thrombolytic medications, microbial fibrinolytic enzymes have gained importance due to minimal side effects, ease of production and are relatively affordable. Marine ecosystem provides a rich environment that is favourable to the growth of many microorganisms. A vast array of secondary bioactive metabolites are formed due to the extensive interaction between the microbial populations in marine habitat.

In this study, actinomycetota have been successfully isolated, screened and the potent isolate was characterized by morphological, biochemical and molecular characterization. The potent bacteria isolated from Rameshwaram water was confirmed to be *A. caeruleus*. Various fibrinolytic protease with high clot lysis activity have been isolated till date. [14] isolated *Streptomyces rubiginosus* from marine sediments, which showed clot lysis activity of 90%, whereas isolate NK60, isolated from marine water exhibited 93.12 ± 0.18% of clot lysis activity, higher activity than Streptokinase, which exhibited 77.14% clot lysis ability. From the sediment sample of Kovalam beach, *Streptomyces lusitanus* was isolated. The purified fibrinolytic fraction showed clot lysis activity of about 65% [16]. *Streptomyces parvulus*, another fibrinolytic protease isolated from lichens of Amazon, showed higher fibrinolytic activity, when the production medium comprised of passion fruit flower [7]. A thermophilic *Streptomyces megasporus* showed in vitro clot-lysis activity at 37℃ [18]. From a hyper-arid environment of Flaming mountain, *Streptomyces fumanus*, was isolated which also exhibited thrombolytic activity [9]. Another *Streptomyces* was isolated from soil sample, which showed better thrombolytic activity than urokinase [17]. A *Streptomyces* VITJS4 from marine saltern, was isolated which exhibited a clot lysis activity of 90% [35]. Another *Streptomyces sp.* CS684 [36] was isolated, which showed good thrombolytic activity. Similarly, from brown marine sponge, *Streptomyces radiopugnans* was isolated, which showed good clot-lysis activity after purification [19]. However, the crude isolate NK60, in this study has shown excellent clot lysis activity, before purification. On purification the potent isolate might exhibit enhanced activity. In the present study, we have only assessed the fibrinolytic activity in vitro, and to address the limitation of our work*, *in vivo studies must be conducted to further determine the role of fibrinolytic protease in treating cardiovascular diseases.

The potent isolate *A. caeruleus* (NK60) is categorized as one of the rare marine actinomycetota [37]. From the seashore sediments, in Weihai *A. cyanogriseus* was isolated, which exhibits antibacterial and cytotoxic activity [38]. Similarly, *A. cyanogriseus* isolated from the sediments of Weihai showed multi-drug resistance reversing activity and the active compound is cyanogramides [39]. From marine sediments of Japan, *Actinoalloteichus* sp. NPS702 was isolated, which exhibited antifungal activity with the bioactive compound Neomaclafungin [40]. In another study, from the Goa beach, marine invertebrate sample was collected, *A. cyanogriseus* was isolated which showed anti-fungal activity against several drug resistant fungal pathogens. The active compound was found to be Caerulomycin A [41]. According to our literature survey, this is the first study that we have heard of on the fibrinolytic activity of rare *A. caeruleus*. Till date, this is one of the few works in which a fibrinolytic protease has been isolated from marine water, as the culturability of microbes in seawater is 0.001–0.10% when compared to soil, which is 0.25%.

Similarly, the synthesis of fibrinolytic enzymes from marine environment by a variety of other microbes, have also been thoroughly investigated by researchers. In a study carried out on marine *Serratia marcescens*, the clot lysis activity was found out to be 38%, which was greater than heparin [42]. Studies on the fibrinolytic potential of purified *Pseudomonas aeruginosa* KU1 revealed that it holds profound thrombolytic activity [43]. In another study carried out on marine bacterium *Bacillus subtilis* ICTF-1, the fibrinolytic enzyme produced had the ability to breakdown the clot effectively [44]. From a marine fungus, *Stachbotrys longispora* FG216 was isolated, which exhibited good fibrinolytic activity [45]. In a study carried out on *Bacillus subtilis subsp. Inaquosorum* isolated from Coringa mangroves, showed 54.70% enhanced fibrinolytic activity after mutagenesis [15]. Thus, various fibrinolytic compounds have been isolated from diverse marine sources, which have demonstrated great potential as thrombolytic agents. Future research however, ought to concentrate on thoroughly evaluating these enzymes as thrombolytic treatments in preclinical and clinical trials in humans and animals.

## Conclusions

In the present study, a fibrinolytic enzyme producing marine actinomycetota *A. caeruleus* was isolated from Bay of Bengal coast. This novel rare actinomycetota represents the first marine bacteria of the genus *Actinoalloteichus* to produce fibrinolytic enzyme. The current study was focused on isolation, screening and characterization of fibrinolytic protease producing marine actinomycetota, where the crude enzyme exhibited a profound fibrinolytic activity, higher than the commercially available thrombolytic drug. However, further purification and optimization studies is also needed to enhance the activity of fibrinolytic enzymes for higher production. In the near future, extensive studies on this enzyme need to be carried out to determine the overall potential for the development of a potent, economical and safe drug with high binding efficacy and fibrin specificity, for the management of cardiovascular diseases, which is the foremost cause of death. Therefore, the study highlights the potential of *A. caeruleus* as a promising candidate for treating thrombotic illness, which may provide new directions in the management of coronary artery disease.

## Data Availability

Genomic Sequence data that supports the finding of this study have been deposited in NCBI with the GenBank accession ID PQ044827.
